# Which Has a Greater Impact on the Recurrence in Young Breast Cancer Patients: Recent Childbirth or Recent Breastfeeding?

**DOI:** 10.1155/2022/5823867

**Published:** 2022-03-31

**Authors:** Caiyun Yan, Jianling Bai, Shengnan Bao, Yiqin Xia, Hao Yu, Yongmei Yin

**Affiliations:** ^1^Department of Biostatistics, School of Public Health, Nanjing Medical University, Nanjing, China; ^2^Department of Medical Oncology, The First Affiliated Hospital of Nanjing Medical University, Nanjing, China

## Abstract

**Purpose:**

This study explored the effects of recent childbirth and recent breastfeeding on the risk of recurrence in patients with postpartum breast cancer (PPBC).

**Materials and Methods:**

A bidirectional cohort study was conducted in the First Affiliated Hospital of Nanjing Medical University. 1013 young female breast cancer patients between May 2003 and October 2019 were enrolled. Breast cancer cases were grouped according to the time between giving birth or weaning and diagnosis. The end point of the analysis was disease-free survival (DFS).

**Results:**

Breast cancer patients diagnosed within 2 years after parturition showed more tumor characteristics that represented poor prognosis and remained at an increased risk for recurrence, even after adjusting for confounding factors (HR = 1.83, *p*=0.035). When the analysis was limited to patients with ER positive or histological grades I and II, they had a higher risk of recurrence. When weaning was used as the grouping node, patients diagnosed within 2 years after weaning did not show a higher risk of recurrence after adjustment, even when analysis was nearly limited to ER-positive patients.

**Conclusion:**

Recent reproductive history is an independent prognostic factor and seems to have a stronger impact on breast cancer with lower malignancy. In addition, the effect of recent childbirth on the recurrence of young breast cancer is significantly stronger than that of recent breastfeeding.

## 1. Introduction

Young breast cancer is particularly aggressive, and its survival has been lagging behind the improvement of the overall level of breast cancer [1], especially in countries with low economic levels [2]. Young women with breast cancer (YWBC) are generally defined as patients aged ≤40 years at diagnosis [3]. The age window for childbearing overlaps with young breast cancer, which becomes the most common tumor during pregnancy and a few years after delivery [2].

According to clinical experience, it has been found that breast cancer patients with a recent history of fertility and lactation tend to have a poor prognosis and therefore the concept of pregnancy-associated breast cancer (PABC) was proposed. However, a number of follow-up studies suggested that the prognosis of breast cancer diagnosed during pregnancy (BCP) was different from that of postpartum breast cancer, and the two were no longer conflated [4, 5]. PPBC referred to breast cancer diagnosed within a few years of the last childbirth, which was defined differently in the study, generally ranging from one to five years after delivery [6–8]. A new meta-analysis of 76 clinical studies showed that PPBC was associated with poor prognosis of breast cancer, and the definition of PPBC should be extended to 6 years after delivery [9]. After adjusting the known prognostic factors such as stage and tumor biological subtype, some studies showed that the prognosis of PPBC patients was still poor [6, 7, 10] and others claimed the poorer prognosis of PPBC was almost fully explained by adverse tumor characteristics [11], while some concluded the strong age effect observed might explain the survival disadvantage in this group [12]. As a consequence, it is necessary to collect more comprehensive clinical data to further clarify whether recent reproductive history is an independent factor affecting the prognosis of patients with PPBC.

Compared with developed countries, the onset age of breast cancer patients in China is younger [13–15] and the situation of childbearing and breastfeeding is also quite different. With the postponement of reproductive age and the introduction of the two-child policy, there will be a significant increase in the number of breast cancer patients with a recent reproductive history. Nevertheless, there are few studies on PPBC in the Chinese population.

In this study, we used two grouping methods to evaluate the tumor characteristics and recurrence risk in women with PPBC and investigated whether the risk of recurrence was related to recent reproductive history in women with different clinical characteristics.

## 2. Materials and Methods

### 2.1. Study Design and Patients

A bidirectional cohort study was conducted on 1013 young female breast cancer patients with stage I–III invasive ductal carcinoma at the First Affiliated Hospital of Nanjing Medical University. Firstly, the medical records of the diagnosis of “breast cancer” or “breast malignant tumor” were searched in the First Affiliated Hospital of Nanjing Medical University between May 1, 2009, and October 30, 2019, including 2057 female breast cancer patients under 40 years of age and 64 aged 41 and 42 years. Female breast cancer patients with stages I–III who were pathologically diagnosed as invasive ductal carcinoma between 20 and 42 years were initially screened. The patient's tumor characteristics were abstracted by retrospective review of the patient files. Then, telephone follow-up was conducted from November 2019 to obtain information about the treatment, recurrence, family history, fertility, and lactation of patients. The reproductive history only asked about the latest pregnancy and childbearing. The telephone interviews included whether the pregnancy occurred before or after the diagnosis of breast cancer, whether it was a complete pregnancy, whether it was an early miscarriage, and the childbearing time of a complete pregnancy. We also asked whether women were diagnosed with breast cancer during lactation and the duration of breastfeeding the last completed pregnancy. The situation of lactation and the cause of weaning were not recorded in the history of breastfeeding. Family history refers only to a family history of breast cancer. They were followed every 12 months until recurrence or the end of the study. Patients with breast cancer who had a miscarriage in the second or third trimester of pregnancy (gestational age ≥12 weeks), who were diagnosed >15 years from the end of the last lactation, who were diagnosed and started treatment during pregnancy, and with other serious diseases were excluded; patients with a history of pregnancy after breast cancer diagnosis were eliminated. Finally, 1013 patients were enrolled in the group of which 166 cases had recurrences at the end of the study, and the average follow-up time was 45.6 months (1–139 months). The time between diagnosis and recurrence was the follow-up time for the patients who had recurrence in the first telephone interview, and the time of follow-up was defined as the time from diagnosis to the end of follow-up for the patients who did not relapse. The definition of follow-up time in the COX model was not the actual follow-up time.

All patients in the group were informed orally and agreed to provide relevant information such as fertility or breastfeeding during telephone follow-up.

### 2.2. Data Collection

We traced the time of the last birth and the end of lactation before the diagnosis of breast cancer and whether there is a history of incomplete pregnancy. Retrospective collection of patients' medical records included demographic characteristics (date of birth, time of diagnosis, family history), tumor characteristics (histological grade, biological subtype, TNM stage, tumor size, lymph node status, ER status, vascular invasion, Ki67), and treatment (surgery, chemotherapy, radiotherapy, endocrine therapy, anti-HER2 therapy). The treatment and recurrence of breast cancer were followed up, including the completion of treatment, the time of the first recurrence, and the location of the first recurrence.

Biological subtypes are defined in the 2013 St. Gallen consensus. The TNM stage was defined according to the AJCC Cancer Staging Manual (8th Edition). The clinical TNM stage was collected for neoadjuvant chemotherapy patients and pathological TNM stage for surgical patients. The tumor size and lymph node status in patients with neoadjuvant chemotherapy were determined according to imaging results or physical examination before chemotherapy and in patients without neoadjuvant chemotherapy according to postoperative pathological results. Histological grade, ER, Ki67, and vascular invasion were reported by immunohistochemistry from surgical specimens of patients with non-neoadjuvant chemotherapy or breast biopsies before neoadjuvant chemotherapy.

The completion of treatment refers to the completion of the scheme recommended by the clinician. Single-target therapy includes trastuzumab, while double-target therapy includes trastuzumab and pertuzumab. Other anti-HER2 treatments were not included.

### 2.3. Exposure

Two types of grouping were performed. Firstly, breast cancer cases were grouped according to time between last childbirth and diagnosis: nulliparous, PPBC <2 (within 2 years after delivery), PPBC 2–5 (during 2–5 years after delivery), PPBC 5–10 (during 5–10 years after delivery), PPBC >10 (over 10 years after delivery). The nulliparous group included patients with a history of early abortion; the PPBC <2 group included patients with breast cancer diagnosed during pregnancy but who began treatment after delivery.

Secondly, according to the interval between the last weaning time and breast cancer diagnosis time, they were divided into nulliparous group, postweaning group I (<2 years after weaning), postweaning group II (2–5 years after weaning), postweaning group III (5–10 years after weaning), and postweaning group IV (10–15 years after weaning). Twelve patients with a lack of breastfeeding information were still grouped according to childbearing time. Patients with breast cancer during lactation were treated after weaning, so they were classified into the postweaning group I.

### 2.4. End Points

The end point of the analysis was disease-free survival (DFS). DFS was calculated in months from the date of diagnosis to the first observation of any recurrence or death. Recurrence was defined as local recurrence, local lymph node recurrence, and distant metastasis confirmed by radiologic imaging and/or pathological results, including invasive ductal carcinoma of the contralateral breast that could not be identified as a primary tumor.

### 2.5. Statistical Analysis

Intergroup differences were assessed by the Chi-square and Fisher's Exact Test (categorical variables) and One-Way ANOVA (continuous variables). Kaplan–Meier analysis was used to assess crude differences in the DFS. Multivariable Cox proportional hazards regression analysis was performed to obtain adjusted risk estimates for DFS, hazard ratios, and 95% confidence. Factors accounted for in the overall analysis included age, stage, and biological subtypes. The proportional hazards assumption was inspected using Schoenfeld residuals. Data were collected and managed using the EDC system (Clinflash), but whether breast cancer was diagnosed during lactation was not recorded in this system. Statistical analyses were performed with SPSS software (SPSS version 20), and analyses were conducted from January 26 to February 2, 2021. A *p* value of less than 0.05 was considered statistically significant.

## 3. Results

### 3.1. Demographic Characteristics, Tumor Characteristics, and Treatment

There were significant differences in the composition of age at diagnosis among the different groups. Among the patients with PPBC <2 years, the number of patients with breastfeeding ≤6 months was more than that of other groups after excluding 19 patients with breast cancer during pregnancy and lactation (*p* < 0.011) (eFigure 1C in the Supplementary Materials). Patients with PPBC <2 years showed more characteristics that represented poor prognosis, such as a high proportion of HER2-enriched, advanced TNM stage and a larger tumor (eFigure 2 in the Supplementary Materials). Nulliparous cases and PPBC >10 cases were more often undergoing breast conservation therapy, but this result was not significant (Table 1).

### 3.2. Difference in Risk of Recurrence in Patients with PPBC

**Figure 1:**
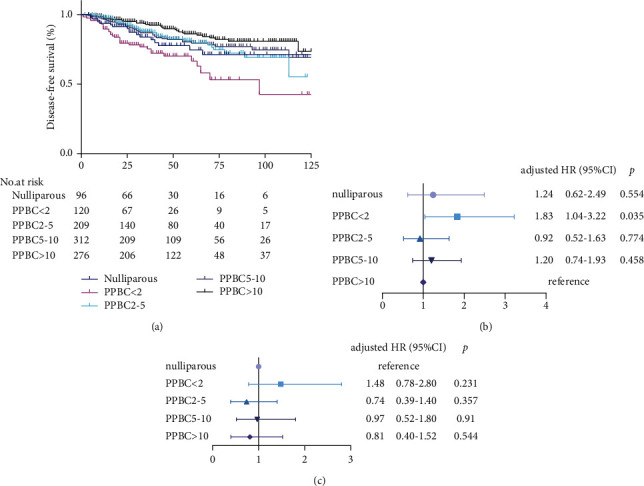
Risk estimate of recurrence in a breast cancer cohort of young women. (a) Kaplan–Meier curves for disease-free survival based on the time since last childbirth, *p* < 0.001; adjusted probability of distance recurrence in cases with PPBC (adjusted for biological subtype, stage, and age of diagnosis); (b) the control group was patients with PPBC >10; (c) the control group was nulliparous cases.

We first compared DFS across the nulliparous, PPBC <2 years, PPBC of 2–5 years, PPBC of 5–10 years, and PPBC>10 years groups. PPBC >10 years served as the control group. KM analysis revealed that the risk of recurrence in patients with PPBC <2 years was significantly higher than that in other groups (Log Rank *p* < 0.001) (Figure 1(a)). Univariate COX regression analysis demonstrated that the risk of recurrence in patients with PPBC <2 years was nearly 2 times higher than that of patients with PPBC >10 years after adjusting for confounding factors (Figure 1(b)).

However, when nulliparous patients served as a control group, neither univariate nor multivariate COX regression analysis showed an increased risk of recurrence in patients with PPBC <2 years (Figure 1(c)).

### 3.3. Effect of Recent Childbirth on Recurrence of Breast Cancer under Different ER Status

Considering that hormones might affect ER status during pregnancy, ER-negative patients accounted for a higher proportion of patients with PPBC <2 years. We conducted a stratified analysis according to the status of ER. The results demonstrated that in ER-positive patients, the risk of recurrence in patients with PPBC <2 years was still high after adjusting for stage and age at diagnosis (HR, 2.28; 95% CI, 1.10 to 4.73; *p*=0.026). However, there was no obvious correlation between DFS and recent childbirth in PPBC patients with negative ER (Table 2).

### 3.4. Effect of Recent Childbirth on the Recurrence of Breast Cancer in Different Histological Grades

**Figure 2:**
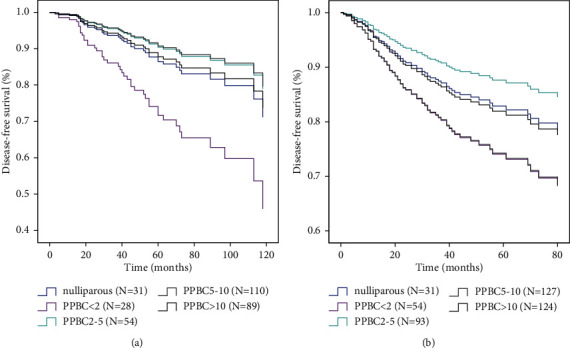
Multivariate COX regression analysis of recurrence risk in breast cancer patients with different histological grades based on the time since last childbirth. (a) Histological grades I and II (PPBC <2 vs. PPBC >10, *p* < 0.1). (b) Histological grade III (PPBC <2 vs. PPBC >10, *p* > 0.1), adjusted for age at diagnosis, TNM stage, and biological subtype. The adjusted recurrence probability function based on the Cox model was generated for each group.

Histological grading is also one of the prognostic factors of breast cancer, but it is not as important as tumor stage and biological subtypes. We carried out a hierarchical analysis of different histological grades. First of all, we compared the differences of DFS in breast cancer patients at different postpartum stages in grades I and II. The results showed that the risk of recurrence in patients with PPBC <2 years was higher than that in patients with PPBC >10 years after adjusting for age, tumor stage, and biological subtype (HR, 2.55; *p*=0.059) (Figure 2(a)). We did not observe a significant difference in DFS in PPBC patients with grade III (Figure 2(b)).

### 3.5. Effect of Lactation on Recurrence of Breast Cancer

**Figure 3:**
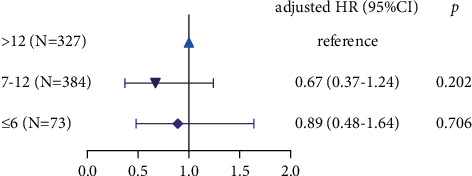
Effect of lactation on the recurrence of breast cancer. Disease-free survival (DFS) is categorized by breastfeeding duration.

Excluding patients with breast cancer during pregnancy and lactation, we found that patients with a breastfeeding duration>12 months had the highest risk of recurrence and patients with a breastfeeding duration of 7–12 months had the lowest risk of recurrence. However, it did not reach statistical significance after adjustment (Figure 3).

### 3.6. Differences of Initial Metastatic Sites in Patients with PPBC

The site of initial metastasis was different among groups, but there was no statistical difference. There were relatively few nonvisceral metastases in nulliparous patients and patients with PPBC <2 years and more lung and liver metastases in patients with PPBC <2 years, which was consistent with their poor prognosis. In addition, there were fewer patients with brain metastasis for the first time and they are not listed separately (eFigure 3 in the Supplementary Materials).

### 3.7. Risk of Recurrence in PPBC Patients Grouped by Weaning Time

The baseline characteristics of patients in each group were similar in the two grouping methods (eTable 1 in the Supplementary Materials). We next compared the differences of DFS in the nulliparous group, postweaning group I, postweaning group II, and postweaning group III, and postweaning group IV was used as control. Univariate COX regression analysis showed that the risk of recurrence in group I was 2.45 times higher than that in group IV, but there was no significant difference between groups after adjustment (95% CI, 0.87 to 2.74; *p*=0.142).Then, we redivided the patients within 5 years after weaning into postweaning group I` (<1 year after weaning) and postweaning group II` (1–5 years after weaning). Following adjustment for confounding factors, the risk of recurrence in group I milarly, we performed stratifie was 1.82 times higher than that in group IV (*p*=0.057) (Table 3). Similarly, we performed stratified analyses by ER status, but no positive results were observed (eTable 2 in Supplementary Materials).

## 4. Discussion

Our results demonstrated that patients with PPBC less than 2 years presented with more adverse tumor characteristics. After adjusting for known prognostic clinical features, the risk of recurrence of breast cancer within 2 years postpartum was still about twice as high as that of patients more than 10 years postpartum and the risk of recurrence was more significant in patients with ER-positive or low-grade cancer. However, when the grouping node was weaning time, the risk of recurrence in PPBC patients with a recent reproductive history was significantly reduced.

We believe that the poor prognosis of breast cancer patients within 2 years after delivery is strongly correlated with TNM stage. Patients with PPBC <2 years are marked by advanced clinical stage, larger tumor size, and increased nodal involvement. This might be attributed to the following reasons: decreased accuracy of breast ultrasound diagnosis during pregnancy or lactation [16], concerns about mammography during pregnancy [17], and women's less self-attention in special periods; all these will lead to the delay of diagnosis. Patients with breast cancer during pregnancy may delay treatment until postpartum because of concerns about fetal safety, although current studies have shown that chemotherapy in the third trimester of pregnancy does not impair neonatal outcome [18, 19]. With the postponement of reproductive age, there will be increasingly patients with PPBC <2 years. Self-examination and ultrasound examination of the breast during pregnancy and at least 2 years after delivery should become routine to improve public awareness so as not to delay the diagnosis of breast cancer.

The biological subtype of breast cancer patients diagnosed within 2 years of their last childbirth showed a higher degree of malignancy (higher proportion of HER2-enriched and ER-negative tumors and a lower proportion of Luminal A tumors), but this cannot be completely explained by the advanced TNM stage. At present, there is no unified conclusion on the theoretical mechanism of the negative effects of recent childbirth on breast cancer recurrence. Mammary gland involution is the most concerned subject in the research on the mechanism of poor prognosis of PPBC [20, 21]. It means that after weaning, or if there is no lactation after delivery, the breast is remodeled and restored from the lactation state to the prepregnancy state [22]. In this process, if a tumor occurs, the growth, invasion, and metastasis of the tumor will be promoted [23–25]. According to the concept of mammary gland involution, the process begins when weaning or not breastfeeding after childbirth. Therefore, weaning as a grouping node can better reflect the role of mammary gland involution in comparing the prognosis of patients with PPBC. Nearly all the research on the prognosis of PPBC considered the birth time as the node, and the results could not directly support that mammary gland involution played a major role in the prognosis of PPBC. Our study used two grouping methods according to childbearing time and weaning time. The results showed that the recent reproductive history or recent lactation history had different effects on the recurrence of PPBC. Taken together, the impact of recent reproductive history was significantly stronger than that of recent breastfeeding. It did not support that mammary gland involution played a major role in the poor prognosis of PPBC. As a result, we speculate that pregnancy and fertility have a greater impact on the prognosis of PPBC than breastfeeding. In addition, the latest literature published in Cancer Discovery has identified that the rate of liver metastasis in postpartum patients diagnosed within 5 years of giving birth was 3.6 times higher than in nulliparous women. The study revealed a previously unknown biology of the rodent liver, weaning-induced liver involution, which provided novel insights into the poor prognosis of women diagnosed with PPBC [26]. Our results showed that the site of initial metastasis was different among groups, but it did not reach statistical significance. Thus, further investigations into the visceral patterns of metastasis in PPBC are necessary. Altogether, the theoretical mechanism of recent fertility leading to an increased risk of breast cancer recurrence still needs to be further explored.

Previous studies have shown that only when the analysis was limited to patients with stage I and II cancer, patients with PPBC less than 10 years were at a significantly increased risk for metastasis [10]. Two other studies showed that the risk of death in patients with PPBC <1 year was significantly increased only in the ER-positive subgroup [6, 11]. Our study showed that the recent reproductive history (nearly 2 years) was an independent prognostic factor for breast cancer, and when the analysis was limited to patients with ER positive or histological grades I or II who exhibited a better prognosis, the prognosis of patients with PPBC <2 years was even worse. According to these results, we believe that the effect of recent childbirth on PPBC recurrence is not strong. When the sample size is small or the follow-up time is short, the effect of recent childbirth on recurrence or death may be masked by tumor biological subtypes and stages in other classifications. Of course, the internal mechanism still needs to be further explored.

Neither the nulliparous patients nor the patients with PPBC >10 years had a recent reproductive history, but in our study, patients diagnosed within 2 years after parturition did not see a worse prognosis compared with the nulliparous patients. We speculate that this may be caused by the following reasons. First, the onset age of breast cancer in China is significantly younger, and the age composition of the nulliparous group is clearly different from that of the developed countries [7, 8, 13]. More than half of them are concentrated before 30 years of age, and the prognosis is relatively poorer. Other unknown factors, such as unrecorded gene mutations, cannot be ruled out. Second, the nulliparous group did not need to follow up the generation time, and the patients recruited into the group were more comprehensive, while the postpartum group cannot be enrolled because some patients or family members did not provide the generation time and the prognosis of the postpartum group might be overestimated. Third, the nulliparous group was quite small relative to the PPBC >10 years.

The duration of breastfeeding may be affected by hormone levels, milk production, lifestyle, and so on [27]. A prospective study in Sweden showed that after adjusting for confounding factors, breast cancer patients who were “overfed” or whose first child was breastfed for more than 12 months had a higher risk of recurrence [27]. Our study showed that the risk of recurrence was the highest in patients who were breastfed for more than 12 months and the lowest in patients with a breastfeeding time of 7–12 months. However, breastfeeding is not an independent prognostic factor for breast cancer recurrence. Baseline data showed that patients with PPBC less than 2 years had the lowest proportion of breastfeeding duration of 7–12 months. This conclusion still needs to be confirmed by further research, such as improving the patient's economy and educational background. In addition, a recent study reported that the mammary glands of mice that abrupt cessation exhibited dense stroma, deposition of collagen, increased expression of ER and PR, higher inflammation, and proliferation. Even at 4 months postpartum, ductal hyperplasia and squamous metaplasia appeared in mice that abrupt interruption of lactation [28]. It is necessary to further determine how gradual and abrupt cessation of weaning affect the breast tissue microenvironment and the prognosis of breast cancer. To better explore the impact of breastfeeding on breast cancer recurrence, follow-up studies should collect more detailed information about breastfeeding, including milk yield, weaning speed, weaning reasons, mastitis time, economic level, and education level.

There are still several imperfections in our research. First, information related to the demographic characteristics of patients is not perfect. There is a lack of information such as BMI index, sports, economic status, and literacy level. Second, this is the result of a single center in a large provincial hospital, and the representativeness of the study population is not nationally representative. Third, the information about the mutation state of BRCA1/2 is missing. Despite these deficiencies, this is one of the few studies based on the Chinese population to assess the risk of recurrence in PPBC patients, including relatively comprehensive breastfeeding information, clinical tumor features, and treatment. It also describes the situation of breastfeeding in patients with PPBC and explores the effect of breastfeeding on breast cancer recurrence.

The risk of recurrence in breast cancer patients diagnosed within 2 years after delivery is significantly higher than that in patients with more than 10 years postpartum. Recent childbirth is an independent prognostic factor with a high risk of recurrence, which has nothing to do with age, tumor stage, and biological subtype, especially in patients with good prognosis. This may become an easily available prognostic index to guide prognosis and treatment. However, recent breastfeeding is not an independent risk factor for the recurrence of breast cancer, suggesting that the recurrence is more related to recent childbirth. The theoretical mechanism of recent childbirth affecting the prognosis of breast cancer needs to be further explored.

## Figures and Tables

**Table 1:** Patient characteristics.

Parity group (1013)	Nulliparous (*N* = 96)	PPBC <2 (*N* = 120)	PPBC 2–5 (*N* = 209)	PPBC 5–10 (*N* = 312)	PPBC >10 (*N* = 276)	*p*
Patients with recurrence, *n* (%)						
Yes	18 (18.8)	33 (27.5)	34 (16.3)	48 (15.4)	33 (12.0)	0.004
No	78 (81.2)	87 (72.5)	175 (83.7)	264 (84.6)	243 (88.0)	
Age at diagnosis, mean (SD), y	30.8 (5.2)	32.1 (3.8)	32.9 (4.2)	35.1 (3.2)	37.5 (2.2)	0.000
Age at diagnosis, *n* (%)						
20–30	54 (56.3)	42 (35.0)	67 (32.1)	28 (9.0)	1 (0.4)	0.000
31–35	17 (17.7)	52 (43.3)	83 (39.7)	131 (42.0)	52 (18.8)	
36–42	25 (26.0)	26 (21.7)	59 (28.2)	153 (49.0)	233 (80.8)	
Breastfeeding duration, *n* (%)						
≤6 months	—	60 (50.0)	86 (41.1)	128 (41.0)	98 (35.5)	0.002
7–12 months	—	43 (35.8)	89 (42.6)	157 (50.3)	157 (56.9)	
≥12 months	—	11 (9.2)	30 (14.4)	25 (8.0)	21 (7.6)	
Missing	—	6 (5.0)	4 (1.9)	2 (0.6)	0 (0.0)	
Family history, *n* (%)						
Yes	6 (6.2)	10 (8.3)	17 (8.1)	18 (5.8)	24 (8.7)	0.872
Year of diagnosis, *n* (%)						
2003–2016	44 (45.8)	58 (48.3)	111 (53.1)	151 (48.4)	159 (57.6)	0.123
2017–2019	52 (54.2)	62 (51.7)	98 (46.9)	161 (51.6)	117 (42.4)	
Histological grade *n* (%)						
I	2 (2.1)	0 (0.0)	2 (1.0)	5 (1.6)	2 (0.7)	0.258
II	33 (34.4)	31 (25.8)	64 (30.6)	114 (36.5)	97 (35.1)	
III	33 (34.4)	57 (47.5)	104 (49.8)	131（42.0）	133 (48.2)	
Missing	28 (29.2)	33 (26.7)	39 (18.7)	62 (19.9)	44 (15.9)	
Nodal involvement, *n* (%)						
N0	49 (51.0)	50 (41.7)	92 (44.0)	164 (52.6)	136 (49.3)	0.154
N1+	42 (43.8)	70 (58.3)	109 (52.2)	144 (46.2)	13 (47.5)	
Missing	5 (5.2)	0 (0.0)	8 (3.8)	4 (1.3)	9 (3.3)	
Estrogen status, *n* (%)						
ER+	64 (66.7)	68 (56.7)	141 (67.5)	218 (69.9)	191 (69.2)	0.097
ER−	32 (33.3)	51 (42.5)	67 (32.1)	90 (28.8)	84 (30.4)	
Missing	0 (0.0)	1 (0.8)	1 (0.5)	4 (1.3)	1 (0.4)	
Surgery type, *n* (%)						
Total mastectomy	52 (54.2)	88 (73.3)	152 (71.8)	219 (70.2)	192 (69.6)	0.133
Breast-conserving	35 (36.5)	29 (24.2)	56 (26.8)	85 (27.2)	84 (30.34)	
Missing	9 (9.4)	3 (2.5)	3 (1.4)	8 (2.6)	0 (0.0)	
Chemotherapy, *n* (%)						
Yes	92 (95.8)	117 (97.5)	204 (97.6)	294 (94.2)	265 (96.0)	0.608
No	2 (2.1)	3 (2.5)	4 (1.9)	12 (3.8)	11 (4.0)	
Missing	2 (2.1)	0 (0.0)	1 (0.5)	6 (1.9)	0 (0.0)	
Radiation therapy, *n* (%)						
Yes	68 (70.8)	80 (66.7)	144 (68.9)	204 (65.4)	189 (68.5)	0.716
No	23 (24.0)	37 (30.8)	61 (29.2)	100 (32.1)	82 (29.7)	
Missing	5 (5.2)	3 (2.5)	4 (1.9)	8 (2.6)	5 (1.8)	
Endocrine therapy, *n* (%)						
Yes	59 (61.5)	59 (49.2)	124 (59.3)	202 (64.7)	186 (67.4)	0.010
No	30 (31.2)	53 (44.2)	70 (33.5)	85 (27.2)	81 (29.3)	
Missing	7 (7.3)	8 (6.7)	15 (7.2)	25 (8.0)	9 (3.3)	
Targeted therapy, *n* (%)						
No	74 (77.1)	86 (71.7)	155 (74.2)	223 (71.5)	230 (83.3)	0.076
Single target	17 (17.7)	28 (23.3)	46 (22.0)	76 (24.4)	40 (14.5)	
Double targets	1 (1.0)	3 (2.5)	5 (2.4)	3 (1.0)	3 (1.1)	
Missing	4 (4.2)	3 (2.5)	3 (1.4)	10 (3.2)	3 (1.1)	

**Table 2:** Risk of recurrence in young breast cancer patients with different ER status.

Parity group	Unadjusted		Adjusted^*∗*^	
	HR (95% CI)	*p*	HR (95% CI)	*p*
ER-positive				
Nulliparous (*N* = 64)	1.63 (0.74–3.61)	0.227	0.92 (0.36–2.39)	0.866
PPBC <2 (*N* = 68)	4.04 (2.13–7.64)	0.000	2.28 (1.10–4.73)	0.026
PPBC 2–5 (*N* = 141)	1.83 (1.00–3.36)	0.051	0.99 (0.48–2.07)	0.985
PPBC 5–10 (*N* = 218)	1.69 (0.95–3.01)	0.076	1.28 (0.70–2.35)	0.430
PPBC >10 (*N* = 191)	1.00		1.00	

ER-negative				
Nulliparous (*N* = 32)	2.31 (1.00–5.34)	0.051	2.36 (0.85–6.56)	0.098
PPBC <2 (*N* = 51)	2.13 (1.00–4.55)	0.051	1.64 (0.69–3.89)	0.260
PPBC 2–5 (*N* = 67)	1.04 (0.46–2.35)	0.919	0.87 (0.34–2.25)	0.769
PPBC 5–10 (*N* = 90)	1.26 (0.62–2.53)	0.524	1.23 (0.57–2.63)	0.599
PPBC >10 (*N* = 84)	1.00		1.00	

^*∗*^Adjusted for age at diagnosis and TNM stage. The control group was patients with PPBC >10.

**Table 3:** Risk of recurrence in PPBC patients grouped by weaning time.

Group	Unadjusted		Adjusted^*∗*^	
	HR (95% CI)	*p*	HR (95% CI)	*p*
Postweaning group IV was used as control				
Nulliparous (*N* = 96)	1.87 (1.04–3.37)	0.037	1.29 (0.63–2.63)	0.488
Postweaning group I (*N* = 161)	2.45 (1.50–4.00)	0.000	1.54 (0.87–2.74)	0.142
Postweaning group II (*N* = 218)	1.86 (1.16–2.98)	0.011	1.18 (0.68–2.06)	0.561
Postweaning group III (*N* = 303)	1.26 (0.78–2.03)	0.343	1.07 (0.64–1.79)	0.787
Postweaning group IV (*N* = 235)	1.00		1.00	

Postweaning group IV was used as control				
Nulliparous (*N* = 96)	1.87 (1.04–3.37)	0.037	1.29 (0.63–2.64)	0.481
Postweaning group I` (*N* = 102)	2.88 (1.68–4.93)	0.000	1.82 (0.98–3.36)	0.057
Postweaning group II` (*N* = 277)	1.85 (1.18–2.91)	0.008	1.16 (0.68–2.00)	0.583
Postweaning group III (*N* = 303)	1.26 (0.78–2.03)	0.343	1.08 (0.65–1.79)	0.781
Postweaning group IV (*N* = 235)	1.00		1.00	

^*∗*^Adjusted for age at diagnosis, TNM stage, and biological subtype.

## Data Availability

The data that support the findings of this study are available from the corresponding author upon request.

## Supplementary Materials

Supplementary Materials eTable 1: baseline characteristics (grouped by weaning time); eTable 2: analysis between parity and ER status for association with DFS when grouped by weaning time; eFigure 1: age at diagnosis and breastfeeding duration; eFigure 2: tumor characteristics; eFigure 3: the site of first recurrence evaluated by parity groups.
