# Supplementary material for: Which Has a Greater Impact on the Recurrence in Young Breast Cancer Patients: Recent Childbirth or Recent Breastfeeding?
Source: Breast J. 2022 Mar 31;2022:5823867. doi: 10.1155/2022/5823867 (PMC9187285; doi:10.1155/2022/5823867)
Supplement: Supplementary Materials — eTable 1: baseline characteristics (grouped by weaning time); eTable 2: analysis between parity and ER status for association with DFS when grouped by weaning time; eFigure 1: age at diagnosis and breastfeeding duration; eFigure 2: tumor characteristics; eFigure 3: the site of first recurrence evaluated by parity groups. [file 5823867.f1.docx]

| **eTable 1. Baseline characteristics (grouped by weaning time)** | | | | | | | |
| --- | --- | --- | --- | --- | --- | --- | --- |
| Parity group（1013） | Nulliparous  （96） | Post-weaning group I  (161) | | Post-weaning group II（218） | Post-weaning group III（303） | Post-weaning group IV（235） | *p* |
| Histologic grade n (%) | | | | | | | |
| Ⅰ | 2(2.1) | 1(0.6) | | 2(0.9) | 5（1.7） | 1（0.4） | 0.322 |
| Ⅱ | 33（34.4） | 48(29.8) | | 65（29.8） | 110（36.3） | 83（35.3） |  |
| Ⅲ | 33（34.4） | 73(45.3) | | 110（50.5） | 126（41.6） | 116（49.4） |  |
| Missing | 28（29.2） | 39（24.2） | | 41（18.8） | 62（20.5） | 35（14.9） |  |
| Biological subtype n (%) | | | | | | | |
| Lumianl A | 8（8.3） | 8（5.0） | | 13（6.0） | 37（12.2） | 24（10.2） | **0.023** |
| Luminal B | 55（57.3） | 87（54.0） | | 130（59.6） | 178（58.7） | 132（56.2） |  |
| Her2-enriched | 12（12.5） | 32（19.9） | | 25（11.5） | 29（9.6） | 20（8.5） |  |
| Triple negative | 18（18.8） | 29（18.0） | | 43（19.7） | 51（16.8） | 49（20.9） |  |
| Missing | 3（3.1） | 5（3.1） | | 7（3.2） | 8（2.6） | 10（4.3） |  |
| TNM stage n (%) | | | | | | | |
| I | 22（22.9） | 34（21.1） | | 53（24,3） | 92（30.4） | 77（32.8） | **0.010** |
| II | 45（46.9） | 61（37.9） | | 92（42.2） | 117（38.6） | 100（42.6） |  |
| III | 17（17.7） | 52（32.3） | | 48（22.0） | 63（20.8） | 40（17.0） |  |
| Missing | 12（12.5） | 14（8.7） | | 25（11.5） | 31（10.2） | 18（7.7） |  |
| Tumor size n (%) | | | | | | | |
| T1 | 34（35.4） | 55（34.2） | | 86（39.4） | 122（40.3） | 118（50.2） | **0.001** |
| T2 | 44（45.8） | 63（39.1） | | 89（40.8） | 130（42.9） | 91（38.7） |  |
| T3 | 6（6.2） | 23（14.3） | | 15（6.9） | 15（5.0） | 6（2.8） |  |
| T4 | 1（1.0） | 4（2.5） | | 2（0.9） | 3（1.0） | 1（0.4） |  |
| Missing | 11（11.5） | 16（9.9） | | 26（11.9） | 33（10.9） | 19（8.1） |  |
| Nodal involvement n (%) | | | | | | | |
| N0 | 49（51.0） | 68（42.2） | | 97（44.5） | 159（52.5） | 118（50.2） | 0.145 |
| N1 | 27（28.1） | 49（30.4） | | 74（33.9） | 81（26.7） | 78（33.2） |  |
| N2 | 11（11.5） | 28（17.4） | | 20（9.2） | 31（10.2） | 21（8.9） |  |
| N3 | 4（4.2） | 15(9.3） | 19（8.7） | | 24（7.9） | 14（6.0） |  |
| Missing | 5（5.2） | （0.6） | 8（3.7） | | 8（2.6） | 4（1.7） |  |
| Estrogen status n (%) | | | | | | | |
| ER+ | 64(66.7) | 97(60.2) | 147(67.4) | | 215(71.0) | 159(67.7) | 0.207 |
| ER- | 32(33.3) | 63(39.1) | 69(31.7) | | 85(28.1) | 75(31.9) |  |
| Missing | 0(0.0) | 1(0.6) | 2(0.7) | | 3(1.0) | 1(0.4) |  |
| Ki67 Labeling Index n (%) | | | | | | | |
| <14% | 15（15.6） | 18(11.2) | 18（8.3） | | 49（16.2） | 31（13.2） | 0.051 |
| 14%-30% | 17（17.7） | 44(27.3) | 50（22.9） | | 70（23.1） | 69（29.4） |  |
| >30% | 55（57.3） | 88(54.7) | 129(59.2) | | 158（52.1） | 111（47.2） |  |
| Missing | 9（9.4） | 11(6.8) | 21（9.6） | | 26（8.6） | 24（10.2） |  |
| Surgery type n (%) | | | | | | | |
| Total-mastectomy | 52（54.2） | 117(72.7) | 159（72.9） | | 212（70.0） | 161（68.5） | 0.095 |
| Breast-conserving | 35（36.5） | 40(24.8) | 56（25.7） | | 84（27.7） | 74（31.5） |  |
| Missing | 9（9.4） | 4(2.5) | 3（1.4） | | 7（2.3） | 0（0.0） |  |
| Chemotherapy n (%) | | | | | | | |
| Yes | 92（95.8） | 157(97.5) | 211（96.8） | | 287（94.7） | 225（95.7） | 0.804 |
| No | 2（2.1） | 4(2.5) | 6（2.8） | | 10（3.3） | 10（4.3） |  |
| Missing | 2（2.1） | 0(0.0) | 1（0.5） | | 6（2.0） | 0（0.0） |  |
| Radiation therapy n (%) | | | | | | | |
| Yes | 68（70.8） | 109(67.7) | 146（67.0） | | 204（67.3） | 158（67.2） | 0.847 |
| No | 23（24.0） | 49(30.4) | 67（30.7） | | 92（30.4） | 72（30.6） |  |
| Missing | 5（5.2） | 3(1.9) | 5（2.3） | | 7（2.3） | 5（2.1） |  |
| Endocrine therapy n (%) | | | | | | | |
| Yes | 59（61.5） | 84(52.2) | 131（60.1） | | 201（66.3） | 155（66.0） | **0.029** |
| No | 30（31.2） | 66(41.0) | 70（32.1） | | 81（26.7） | 72（30.6） |  |
| Missing | 7（7.3） | 11(6.8) | 17（7.8） | | 21（6.9） | 8（3.4） |  |
| Targeted therapy n (%) | | | | | | | |
| No | 74（77.1） | 113(70.2) | 165（75.7） | | 221（72.9） | 195（83.0） | 0.130 |
| Single-target | 17（17.7） | 38(23.6) | 49（22.5） | | 69（22.8） | 34（14.5） |  |
| Double-target | 1（1.0） | 5(3.1) | 3（1.4） | | 3（1.0） | 3（1.3） |  |
| Missing | 4（4.2） | 5(3.1) | 1（0.5） | | 10（3.3） | 3（1.3） |  |

| **eTable 2. Analysis between parity and ER status for association With DFS when grouped by weaning time** | | | |
| --- | --- | --- | --- |
| Group | | Unadjusted  HR (95%CI) *p* | Adjusted*  HR (95 % CI) *p* |
| ER-positive | | | |
| Nulliparous(N=64) | 1.53（0.68—3.43）0.304 | | 0.94（0.35—2.51）0.900 |
| Post-weaning group I(N=97) | 2.69（1.43—5.07）0.002 | | 1.47（0.69—3.13）0.317 |
| Post-weaning group II(N=147) | 1.96（1.06—3.61）0.032 | | 1.12（0.54—2.32）0.768 |
| Post-weaning group III(N=215) | 1.35（0.73—2.50）0.344 | | 1.12（0.58—2.14）0.736 |
| Post-weaning group IV(N=159) | 1.00 | | 1.00 |
|  | ER-negative | |  |
| Nulliparous(N=32) | 2.42（1.02—5.76）0.046 | | 1.95（0.65—5.90）0.236 |
| Post-weaning group I(N=63) | 1.78（0.81—3.92）0.152 | | 1.26（0.51—3.15）0.618 |
| Post-weaning group II(N=69) | 1.48（0.69—3.21）0.318 | | 1.09（0.44—2.69）0.847 |
| Post-weaning group III(N=85) | 1.21（0.57—2.55）0.621 | | 1.13（0.49—2.61）0.771 |
| Post-weaning group IV(N=75) | 1.00 | | 1.00 |

*Adjusted for age at diagnosis and TNM stage.

**eFig. 1** **Age at diagnosis and breast-feeding duration**

A. Age at diagnosis（*p*<0.001）. B. Average lactation time and SD value of patients with PPBC（*p*=0.138）C. Breast-feeding duration of patients with PPBC(*p*=0.011). B, C Breast cancer during pregnancy and lactation was not included.

**eFig. 2. Tumor characteristics**

A.TNM stage（*p*=0.014）B. Tumor size（*p*=0.001）C.Ki67 Labeling Index（*p*=0.034） D. Biological subtype（*p*=0.013）

**eFig. 3. The site of first recurrence evaluated by parity groups**

The contralateral breast, local breast, soft tissue, bone, lymph nodes and chest wall were non-visceral metastasis. More than one site of the first recurrence was multiple metastasis. Lung, liver and brain are the first single metastatic sites. (*p*>0.05)
